# Melanopsin in the human and chicken choroid

**DOI:** 10.1016/j.exer.2024.110053

**Published:** 2024-08-14

**Authors:** Christian Platzl, Alexandra Kaser-Eichberger, Andrea Trost, Clemens Strohmaier, Richard Stone, Debora Nickla, Falk Schroedl

**Affiliations:** a Center for Anatomy and Cell Biology, Institute of Anatomy and Cell Biology -Salzburg, Paracelsus Medical University, Salzburg, Austria; b Dept. of Ophthalmology and Optometry, Paracelsus Medical University, Salzburg, Austria; c Department of Ophthalmology and Optometry, Johannes Kepler University, Linz, Austria; d Department of Ophthalmology, University of Pennsylvania Perelman School of Medicine, Philadelphia, USA; e Dept. of Biomedical Sciences and Disease, The New England College of Optometry, Boston, USA

**Keywords:** Choroid -innervation, Nervous system, Emmetropization, Blood flow –circadian rhythms- OPN4

## Abstract

The choroid embedded in between retina and sclera is essential for retinal photoreceptor nourishment, but is also a source of growth factors in the process of emmetropization that converts retinal visual signals into scleral growth signals. Still, the exact control mechanisms behind those functions are enigmatic while circadian rhythms are involved. These rhythms are attributed to daylight influences that are melanopsin (OPN4) driven. Recently, OPN4-mRNA has been detected in the choroid, and while its origin is unknown we here seek to identify the underlying structures using morphological methods.

Human and chicken choroids were prepared for single- and double-immunohistochemistry of OPN4, vasoactive intestinal peptide (VIP), substance P (SP), CD68, and α-smooth muscle actin (ASMA). For documentation, light-, fluorescence-, and confocal laser scanning microscopy was applied. Retinal controls proved the reliability of the OPN4 antibody in both species.

In humans, OPN4 immunoreactivity (OPN4-IR) was detected in nerve fibers of the choroid and adjacent ciliary nerve fibers. OPN4+ choroidal nerve fibers lacked VIP, but were co-localized with SP. OPN4-immunoreactivity was further detected in VIP+/SP + intrinsic choroidal neurons, in a hitherto unclassified CD68-negative choroidal cell population thus not representing macrophages, as well as in a subset of choroidal melanocytes. In chicken, choroidal nerve fibers were OPN4+, and further OPN4-IR was detected in clustered suprachoroidal structures that were not co-localized with ASMA and therefore do not represent non-vascular smooth-muscle cells. In the choroidal stroma, numerous cells displayed OPN4-IR, the majority of which was VIP-, while a few of those co-localized with VIP and were therefore classified as avian intrinsic choroidal neurons. OPN4-immunoreactivity was absent in choroidal blood vessels of both species.

In summary, OPN4-IR was detected in both species in nerve fibers and cells, some of which could be identified (ICN, melanocytes in human), while others could not be classified yet. Nevertheless, the OPN4+ structures described here might be involved in developmental, light-, thermally-driven or nociceptive mechanisms, as known from other systems, but with respect to choroidal control this needs to be proven in upcoming studies.

## 1. Introduction

Melanopsin (OPN4) is a widely distributed photopigment first localized to melanocytes of *Xenopus laevis* and is the opsin underlying the intracellular migration of dermal melanocytes (Provencio et al., 1998). Its subsequent localization in numerous retinal cell types includes a group of intrinsically-photosensitive retinal ganglion cells (ipRGCs), some of which project to the suprachiasmatic nucleus (SCN) and regulate circadian entrainment (Gooley et al., 2001; Hannibal et al., 2002; Provencio et al., 2000). Among other visual functions mediated by OPN4 are detection of light intensity, the pupil light response, and contrast and pattern detection (Allen et al., 2019; Brown et al., 2012; Spitschan, 2019). Besides retinal neurons, OPN4 also localizes to pineal gland (Bailey and Cassone, 2005; Chaigne et al., 2024), vascular cells (Barreto Ortiz et al., 2018; Sikka et al., 2014), iris (Tsuchiya et al., 2017; Xue et al., 2011), cornea (Delwig et al., 2018), retinal pigment epithelium (RPE) (Peirson et al., 2004), lens epithelial cells (Alkozi et al., 2017), trigeminal neurons projecting to the eye (Delwig et al., 2018; Matynia et al., 2016) and skin melanocytes (Lima et al., 2006); for a more recent review of opsins and opsin action please see (Andrabi et al., 2023).

Recently, we identified the expression of OPN4 mRNA in the choroid (Stone et al., 2020), a vascular structure behind the RPE. Classically, the choroid supplies nutrients and oxygen to the outer retina (Linsenmeier and Padnick-Silver, 2000) and may modulate ocular or retinal temperature (Parver et al., 1980). Pathology in the choroid also may contribute to various outer retinal diseases, including macular degeneration (Farazdaghi and Ebrahimi, 2019; Fragiotta et al., 2022). In both animals and humans, the choroid is a dynamic and multi-functional structure. The choroid undergoes a diurnal oscillation in thickness, likely under circadian control (Nickla et al., 1998a, 2002, 1998b). The choroidal thickness and its fluctuations seemingly participate in the mechanisms of emmetropization and refractive error development (Nickla and Wallman, 2010), but other possible functions for these dynamic changes are not known (Chakraborty et al., 2018; Nickla et al., 1998c, 2002). Supporting the notions that OPN4 may participate in the underlying mechanisms mediating emmetropization or ametropias, the mRNA levels for OPN4 oscillate during the day, and these oscillations are perturbed in both retina and choroid of chick eyes developing myopia or hyperopia (Stone et al., 2020). Furthermore, mutant mice lacking OPN4 from either an OPN4-knockout or from non-functioning ipRCGs show abnormal refractive development, but the inability to distinguish the role of OPN4 from functioning ganglion cells in this model makes interpretation difficult (Chakraborty et al., 2022). Given the potential for varied and complex ocular functions for OPN4 in the choroid, we here used immunohistochemistry in human and chick choroids to identify the cells and/or structures expressing OPN4.

## 2. Material and methods

### 2.1. Tissue origin

Tissues from 6 human donors (age 63–73 years, both sexes; post mortem time 13–20 h) were obtained from the Cornea Bank of the Department of Ophthalmology, or from the body donor program of the Center for Anatomy and Cell Biology, Institute of Anatomy and Cell Biology, Salzburg, both at the Paracelsus Medical University, Salzburg, Austria. Tissues were retrieved also in full accordance with the Declaration of Helsinki and approved by the local ethics committee (415-EP/73/775–2018 and EK1012/2019). Mature chicken eyes (14 weeks of age; White Leghorn; n = 3) were obtained from a local poultry farm. These eyes showed no obvious signs of pathological alterations.

### 2.2. Tissue preparation

The eyes were opened at the ora serrata and fixed by immersion in 4% paraformaldehyde (PFA) diluted with phosphate buffered saline (PBS, 1 h at room temperature, RT). After a rinse in PBS (12 h) and transfer into PBS containing 15% sucrose (12 h at 4 °C), tissues were frozen at −80 °C using liquid nitrogen-cooled methylbutane and stored at −20 °C for further processing. Before sectioning, the tissue was embedded in tissue embedding medium (Fisher Scientific GmbH, Vienna, Austria). Tissue sections (14 μm thickness) were prepared in a cryostat (HM 550, Microm, Walldorf, Germany), either as horizontal sections from flat-mount preparations (Schrödl et al., 2000) or cross sections, and were collected on adhesion slides (Superfrost Plus; Thermo Scientific, Vienna, Austria) mainly from temporal and superficial to stromal origins. Slides were air-dried for 1 h at RT and specimens were then rehydrated in tris-buffered saline (TBS; Roth, Karlsruhe, Germany) for 5 min followed by an antigen retrieval-protocol using citrate buffer at pH 6 at 65 °C for 30 min. Following a 5 min rinse in TBS, slides were incubated for 1h at RT in TBS containing 10% donkey serum (Sigma-Aldrich, Wien, Austria), 1% bovine serum albumin (BSA; Sigma-Aldrich), and 0.5% Triton X-100 (Merck, Darmstadt, Germany). After a rinse (three times 5 min), slides were incubated for single or double immunohistochemistry of the markers listed in Table 1 in TBS, containing 1% BSA and 0.5% Triton X-100, 12 h at RT, followed by another rinse in TBS (three times 5 min). Binding sites of primary antibodies were visualized by corresponding Alexa488-or Alexa555-tagged antisera (1:1000; Invitrogen, Karlsruhe, Germany) in TBS, containing 1% BSA and 0.5% Triton X-100 (1 h at RT, light exclusion) followed by another rinse in TBS (three times 5, light exclusion). Some of the slides received an additional nuclear staining using 4’,6-Diamidino-2-phenylindol dihydrochloride (DAPI). For that, slides were incubated for 10 min (1:4000, stock 1 mg/ml, VWR, Vienna, Austria, light exclusion) followed by a rinse in PBS (three times 5 min, light exclusion). All specimens were finally embedded in TBS-glycerol (1:1 at pH 8.6). All antibodies used were generated against human epitopes. Human and chicken retina served as positive controls; negative controls were performed by omission of the primary antibodies/antisera during incubation and resulted in absence of immunoreactivity. For simplicity, the gene name “OPN4” will be used as abbreviation for melanopsin throughout the manuscript.

### 2.3. Documentation

In order to document single- and double-labeled immunohistochemistry, a confocal laserscanning unit (Axio ObserverZ1 attached to LSM710, Zeiss, Göttingen, Germany; × 20 dry or × 40 and × 60 oil immersion objektive lenses, with numeric apertures 0.8, 1.30, and 1.4, respectively; Zeiss) was used. Sections were imaged using the appropriate filter settings for Alexa555 (568 nm excitation, channel 1, coded red), Alexa488 (488 nm excitation, channel 2, coded green) and DAPI (345 nm excitation, coded white) and up to four channels were detected simultaneously. Alternatively, a fluorescence microscope (Axio Imager M2 equipped with AxioCam HRc and ApoTome 2 Unit; Zeiss, Göttingen, Germany) was used to document immunofluorescence in chicken specimens. In all experiments, identical camera/laser settings were used for acquisition of the corresponding negative controls. In human specimens, a donor-dependent variation in signal intensity was observed.

## 3. Results

### 3.1. Retinal controls

**Fig. 1.**
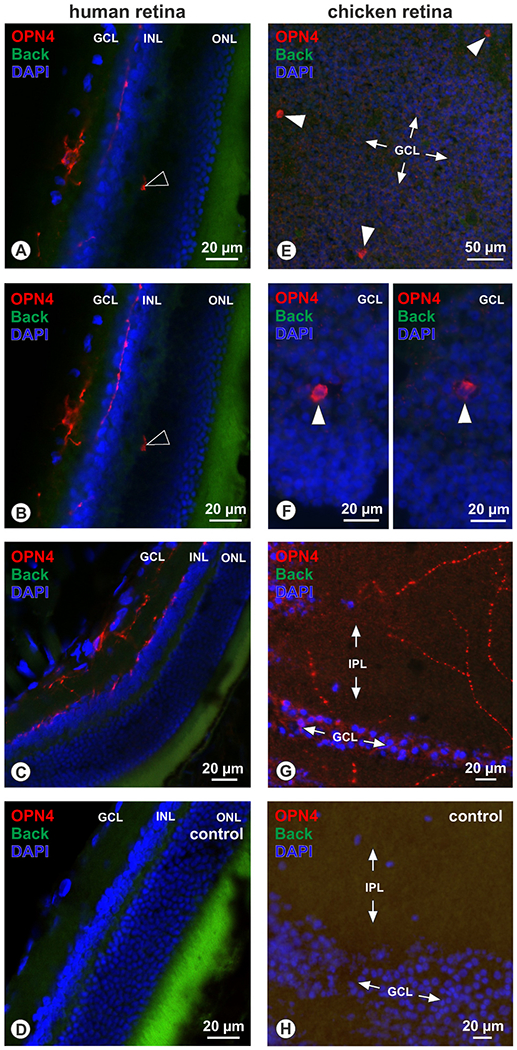
Retinal Controls In cross sections of human retina (A to D), OPN4-immunoreactivity (red) was detected in a few cells of the GCL, with their processes reaching into INL (A, B; consecutive optical section of the confocal microscope with z-axis = 1 μm), while in other sections OPN4+ fibers were detected that terminated at the inner border of the INL (C). Note also an OPN4 immunoreactive process in the outer plexiform layer (open arrowhead in A, B). Immunoreactivity, was absent in corresponding negative controls (D) (green = background fluorescence, Back; blue: DAPI nuclear stain; GCL = ganglion cell layer; INL = inner nuclear layer; ONL = outer nuclear layer).In sections of flatmount preparations of the chicken retina (E to H), OPN4 immunoreactivity (red) was detected in few cells of the GCL (filled arrowheads; E, F, with F representing a magnification of the situation in E), and further in nerve fibers forming boutons within the IPL (G). Note that the different regions in these flatmount sections, like GCL in figure G and H, arise due to tissue folds and oblique cutting. Immunoreactivity was absent in corresponding negative controls (H).

Human and chicken retinas served as positive controls for the reliability of the OPN4 antiserum. In human retinas (Fig. 1A–D), a few cells in the ganglion cell layer (GCL) that had processes extending into the inner nuclear layer (INL) were immunoreactive for OPN4 (Fig. 1A–C), while corresponding negative controls lacked immunoreactivity (Fig. 1D). In chicken retinas, flat-mount preparations (Fig. 1E–H) revealed OPN4-immunoreactivity in occasional cells in the GCL (Fig. 1E and F), and also in distinct bouton-forming neuronal processes in the inner plexiform layer (IPL; Fig. 1G). Negative controls lacked OPN4 immunoreactivity in both layers (Fig. 1H).

### 3.2. Human choroid

#### Nerve Fibers:

**Fig. 2.**
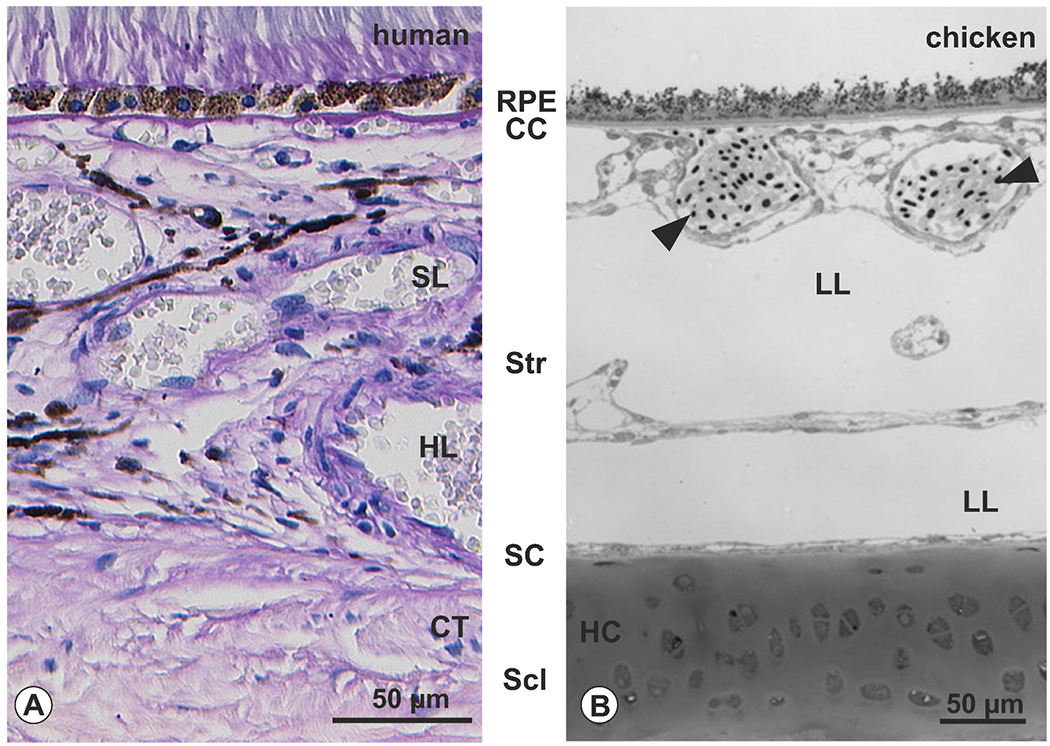
Cross sections for result assignment In order to facilitate orientation in the species investigated, choroidal cross sections in human (A, standard PAS-staining) and chicken (methylene blue) are provided, and corresponding resulting areas of the following figures indicated. Scl: sclera; SC: suprachoroid; Str: choroidal stroma; CC: choriocapillaris; RPE: retinal pigment epithelium. Note that within the choroidal stoma in humans, Haller’s (HL) and Sattler’s layer (SL) can be discriminated based on blood vessel diameters (Zhang et al., 2024), whereas in birds lymphatic lacunae (LL) exist (De Stefano and Mugnaini, 1997; Ostrin et al., 2023). Note also that the inner portion of the avian sclera consists of hyaline cartilage (HC), whereas in human, it consists of connective tissue (CT), and further that avian erythrocytes do contain nuclei (arrowheads). (For interpretation of the references to colour in this figure legend, the reader is referred to the Web version of this article.)

**Fig. 3.**
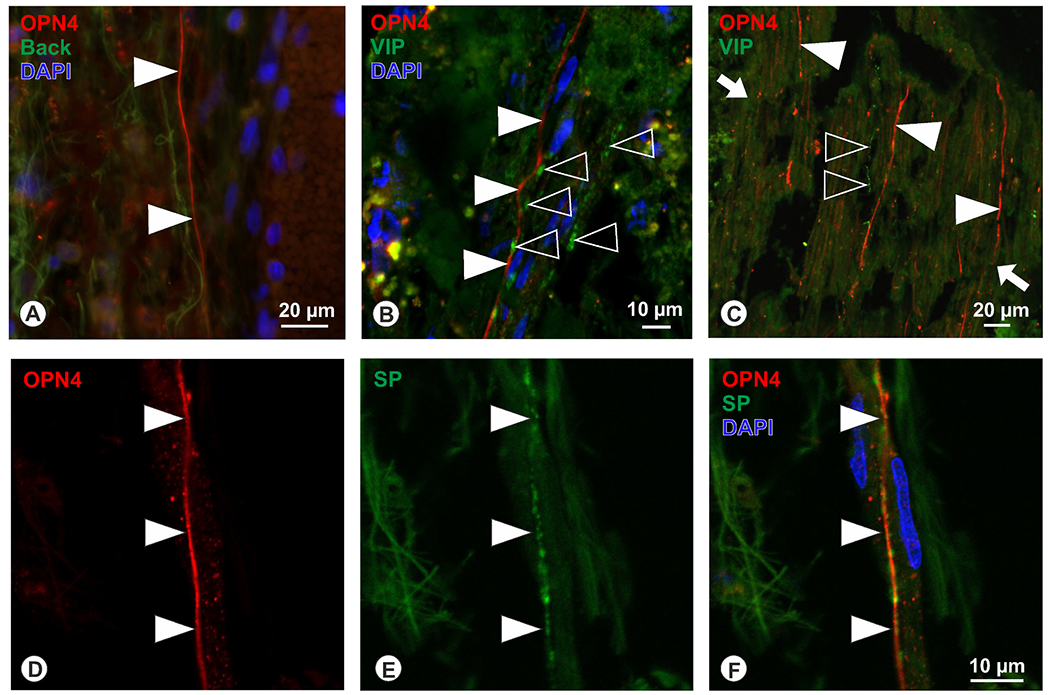
Nerve fibers in the human choroid In the human choroid, OPN4-immunoreactive nerve fibers (red) with rather uniform diameter (A, filled arrowheads), or forming boutons (B, filled arrowheads) were detected in the choroidal stroma in between Haller’s and Sattler’s layer, or in nerve strands of the ciliary nerve (C, filled arrowheads) in between suprachoroid and sclera. These nerve fibers were not co-localized with VIP (green, open arrowheads in B, C), as seen in single optical sections of the confocal microscope (arrows in C indicate the extension of a ciliary nerve strand on the suprachoroid; dark spots here are caused due to bending of the nerve and the confocal imaging on the tissue surface). Co-localization experiments with OPN4 (red, D) and SP (green, E) revealed an overlap of both markers (F) in nerve fibers (arrowheads) of the choroidal stroma (Blue: DAPI nuclear stain; single optical section of the confocal microscope).

OPN4 immunoreactivity was detected in nerve fibers within the choroidal stroma in between Haller’s and Sattler’s layer (for better orientation in human and chicken choroid, please see Fig. 2), some of which had uniform diameters (Fig. 3A), and some that formed boutons (Fig. 3B). OPN4 was also found in some ciliary nerves, identified by their large diameters and location within the suprachoroid (Fig. 3C). These OPN-positive fibers were negative for VIP (Fig. 3C), and positive for SP (Fig. 3D–F).

#### Cells:

**Fig. 4.**
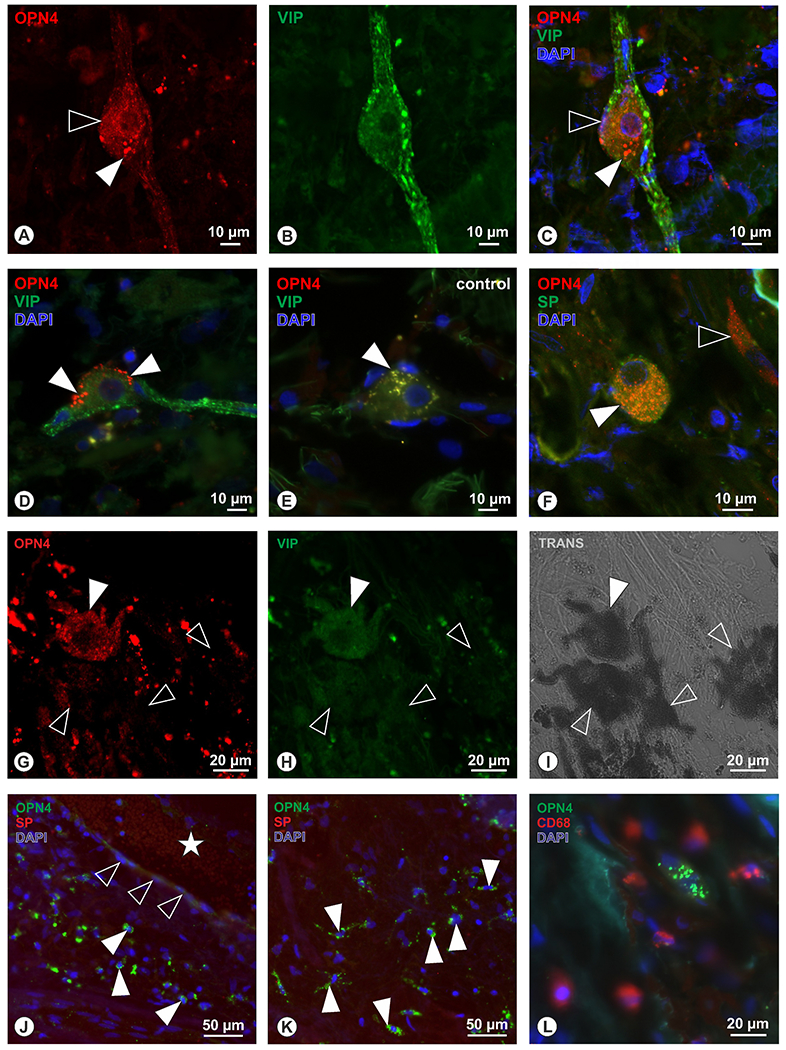
Cells in the human choroid In the choroidal stroma between Haller’s and Sattler’s layer, OPN4+ cells (red, A) were detected, that were co-localized with VIP (green, B). While OPN4 immunoreactivity displayed a fine granular cytoplasmatic reaction pattern (open arrowhead in A, C), additionally a prominent polar accumulation of OPN4-immunoreactivity with larger granule-like appearance was observed (arrowhead in A, C). Larger OPN4+ granules were often seen without the fine granular cytoplasmatic pattern (D, arrowheads). The larger OPN4+ granules were not identical with lipofuscin granules often present in intrinsic choroidal neurons, as detected by their green-yellow autofluorescent signal in negative controls (E, arrowhead). These larger OPN4-immunoreactive granules with polar accumulation (A, C, D) may be degradation products. In co-localization experiments with SP (F), OPN4+ cells (red) displayed also SP-immunoreactivity (green, filled arrowhead), as indicated by orange granules. By size, shape, and immunoreactivity these aforementioned cells were identified as ICN, while other cells were small and elongated cells displaying OPN4-immunoreactivity only (open arrowhead in F) and could not be classified yet.G to I: Other rather large irregular shaped cells in the choroidal stroma were detected with faint OPN4 immunoreactivity (G, filled arrowhead) lacking VIP immunoreactivity (H), but contained melanin granules, as seen in transmitted light mode of the confocal microscope (I), while the majority of melanin-containing cells (open arrowheads in G to I) lacked OPN4-immunoreactivity. J: While some OPN4+ cells (F) were reminiscent of endothelial cells, in sections with clearly a identified blood vessel (J, star displays lumen of blood vessel), adjacent endothelial cells were OPN4 and SP negative (J, open arrowheads). Within the choroidal stroma numerous other small irregular shaped cells displayed OPN4-immunoreactivity (green; filled arrowheads in J, K) but lacked SP immunoreactivity. While these small cells may have represented macrophages, co-localization experiments (L) with OPN4 (green) and the macrophage marker CD68 (red) could not demonstrate a co-localization of both markers.

OPN4-immunoreactivity with a fine granular appearance was found in the cytoplasm of VIP-positive intrinsic choroidal neurons (Fig. 4A and B) within the choroidal stroma; some of these neurons also contained larger intensely stained granules (Fig. 4C). Some of these double-labeled neurons that lacked the fine granularity contained large, intensely-labeled OPN- granules whose distribution was confined to the soma periphery (Fig. 4D). This “polar” localization differed from that of lipofuscin granules in negative controls (green fluorescence in Fig. 4E) based on their size, labeling intensity, intracellular localization and fluorescence signal at 488 nm excitation. A second type of OPN-positive cells co-localized with Substance-P (SP) (Fig. 4F). Within the choroidal stroma, a third set of small, elongated OPN-positive cells resembling endothelial cells were negative for both VIP and SP (Fig. 4F), however, in our hands, OPN-labeling was not seen in any of the cells lining clearly defined blood vessels, so their identity as endothelium is unsupported. Finally, we observed large, irregularly shaped cells with fine granular OPN4 immunoreactivity (Fig. 4G) that did not co-localize with VIP (Fig. 4H) within the stroma. Trans-illumination with a confocal microscope identified melanin granules in these cells (Fig. 4I), which comprised about 10% of all the melanin-containing cells.

As noted above, the endothelial cells of vessel lumens were negative for OPN4 (Fig. 4J). However, in the immediate vicinity were numerous small, irregular-shaped OPN4-positive cells that did not co-localize with SP (Fig. 4J and K). While these cells had the morphology of choroidal macrophages, they were negative for the macrophage marker CD68 (Fig. 4L).

### 3.3. Chicken choroid

**Fig. 5.**
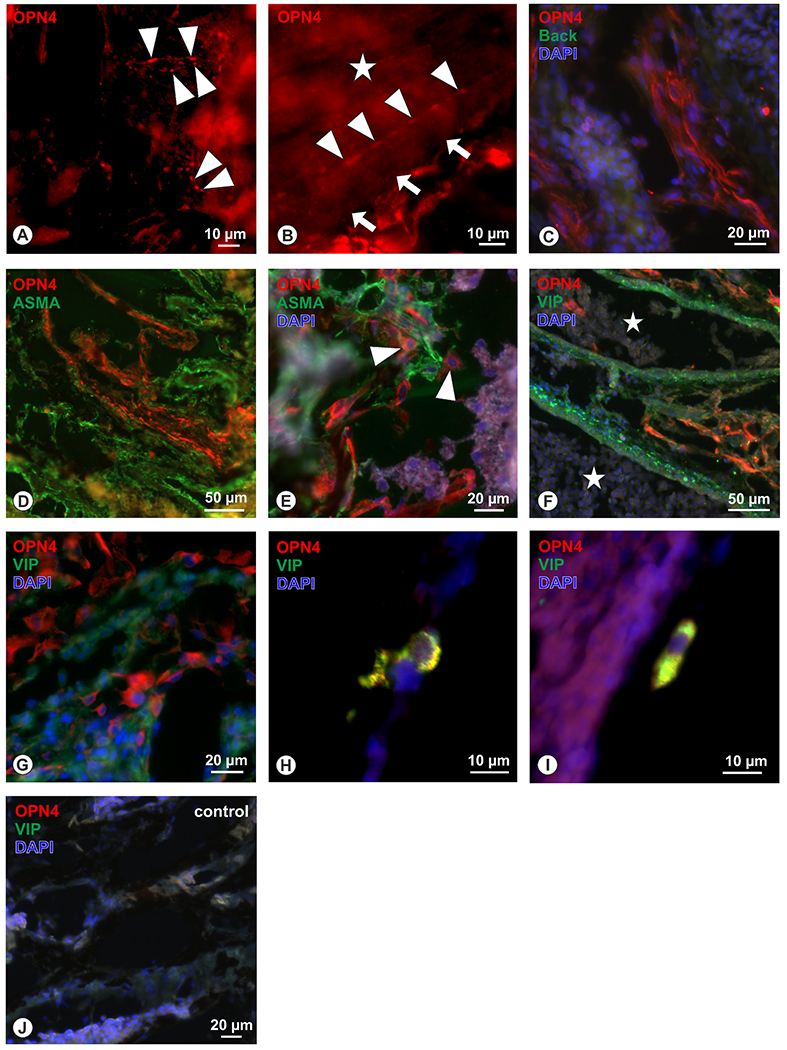
OPN4 in the chicken choroid In the chicken choroid, nerve fibers forming boutons were immunoreactive for OPN4 (red) within the choroidal stroma (A, arrowheads) and, B) in nerve strands (arrowheads) of choroidal nerves (star; border of the nerve strand is indicated by arrows and it extends beyond left upper margin of the micrograph). In certain layers of the suprachoroid, a strong and clear increase of the OPN4 immunoreactivity was detected (C), but the nature of these underlying structures was not clear. Co-localization OPN4 (red) and ASMA (green) revealed no overlap of the two markers (D). While some OPN4+ cellular elements within the stroma were seen (E, arrowheads), these were also ASMA negative. Further, these OPN4+ structures (red) were not related to choroidal blood vessels (F) as identified by the VIP-immunoreactive (green) perivascular plexus adjacent to the lumen of choroidal blood vessels (stars). The majority of identified (G) OPN4+ cells (red) were lacking VIP-immunoreactivity (green), while a few cells were identified (H, I) where OPN4 and VIP co-localized and these were determines as ICN. In corresponding negative controls, OPN4 and VIP immunoreactivity was absent (J).

OPN4 immunoreactivity was detected in individual bouton-forming nerve fibers within the choroidal stroma (Fig. 5A), as well as within large bundles of nerves (Fig. 5B). In the suprachoroid, clustered OPN4-positive structures reminiscent of non-vascular smooth muscle cells (Stübinger et al., 2010) were seen (Fig. 5C), but double-labelling showed no co-localization with alpha-smooth muscle actin (ASMA) (Fig. 5D). Deeper in the stroma, and not associated with vessels (Fig. 5F), were cluster-forming OPN4-positive cells resembling avian intrinsic choroidal neurons (ICNs); these were irregular in shape or elongated, and had large round nuclei. A small number of these were positive for VIP (Fig. 5H and I), while the majority were VIP-negative (Fig. 5F and G); they were all negative for ASMA (Fig. 5E). Immunoreactivity was absent in all negative controls (Fig. 5J).

## 4. Discussion

We used immunohistochemistry in human and chicken choroids to identify structures/cells expressing OPN4. OPN4-immunoreactivity was observed in nerve fibers, intrinsic choroidal neurons, and unidentified small cells in both species. In human choroid, melanin-containing melanocytes were positive for OPN4. In chicken only, staining was found in clustered structures that were reminiscent of non-vascular smooth muscle, but negative for alpha-smooth actin, precluding identification as such.

Our affinity-purified polyclonal antibody was directed against a synthetic peptide of rat OPN4. This antibody does not discriminate between the two OPN4 isoforms observed in non-mammalian species such as the chicken, namely OPN4x and OPN4m whereas in mammals OPN4m is expressed only (Bailey and Cassone, 2005; Bellingham et al., 2006). Nevertheless, our control experiments revealed OPN4-immunopositivity for a subset of ganglion cells in chicken retina, and in both species, for neuronal processes, and these results were favorable in line with earlier observations of ipRGCs (Lax et al., 2019; Nasir-Ahmad et al., 2019; Verra et al., 2011) using different antibodies.

### 4.1. Human choroid

The human choroidal stroma contained OPN4-labeled nerve fibers, some of which we identified as efferents of the ciliary ganglion, based on diameter and location. The various other OPN4-positive fibers are likely sensory afferents of the trigeminal ganglion, some of which might innervate anterior eye (cornea and iris) and run through the ciliary ganglion (Delwig et al., 2018; Xue et al., 2011), consistent with their known localization of both OPN4 and SP (Quartu et al., 1992; Stone and Kuwayama, 1985). In addition, the OPN4-positive fibers that co-localize with SP might originate from ICNs, a sub-population of which express SP (de Hoz et al., 2008). Because the bouton-forming OPN4-positive fibers were negative for VIP, the pterygopalatine ganglion as a site of origin seems unlikely (Cuthbertson et al., 1997; Stübinger et al., 2010; Uddman et al., 1980).

We found several morphologically distinct cell types that labeled for OPN4. One was identified as intrinsic choroidal neurons based on morphology and co-labelling for VIP (Bergua et al., 1993; Flugel et al., 1994; Miller et al., 1983). These ICNs showed two distinct labelling patterns: a) diffuse and finely grained; and b) intensely-labeled, large granules often localized to the soma periphery, i.e. with polar arrangement. We speculate that these larger granules might be a melanopsin degradation product, however, the process by which melanopsin is deactivated and degraded is not known (Contreras et al., 2021). Still, these granules were clearly discernible from the lipofuscin granules present in aging neurons (Brehmer et al., 2004; Ottis et al., 2012) and also ICN (May et al., 2004; Schrodl et al., 2015a, 2015b; Summers et al., 2020). Another cell type contained small, irregularly shaped cells morphologically similar to macrophages; however, the lack of labeling for CD68 largely precludes this identification since CD68 can be well used as a screening marker for macrophages (Ferenbach and Hughes, 2008). As such, it has been used earlier in the choroid (Schroedl et al., 2008; Summers et al., 2020) while also alternative macrophage markers have been applied, (e.g., IBA1; (McLeod et al., 2016). Future studies are required for a further thorough classification of immune cells potentially involved. A third cell type comprised a small population identified as melanocytes, consistent with earlier reports of OPN4-containing melanocytes in cell cultures of chicken (Lima et al., 2006), mouse (de Assis et al., 2016) and human skin melanocytes (Kusumoto et al., 2020). Within the limits of our methods, OPN4 immunoreactivity was absent in the walls of blood vessels, despite earlier reports of OPN4 in vascular systems (Sikka et al., 2014; Stone et al., 2020).

### 4.2. Chicken choroid

Some of the OPN4 positive fibers in chicken choroid appeared morphologically similar to those in human choroid. We also found OPN4-immunoreactive structures that resembled non-vascular smooth muscle, but they were negative for α–smooth-muscle actin. These cells might be specialized fibroblasts or be part of the walls of the choroidal lacunae; the lack of appropriate markers for such precludes identification (Summers et al., 2020). Similar to human choroid, blood vessel walls were negative for OPN4. However, there was an abundance of OPN4-immunoreactive cells in the choroidal stroma of chicken, some of which appeared to be neurons. A number of these were positive for VIP, identifying them as ICNs (Stübinger et al., 2010) while the nature of the VIP-negative subpopulation needs to be established. Nothing could be concluded regarding melanocytes, because the strain we used lacked melanin in the choroid and further studies in mammals are necessary to transfer our findings in an appropriate animal model. The identity of the other cells awaits further study. Possibilities include fibroblasts (Campbell et al., 2014), immune-cells (McMenamin et al., 2019), or other reported unknown cells in choroidal stroma and lamina fusca (Petrea et al., 2018; Summers et al., 2020). While markers for these cells are complex or not well-established, scRNA applications in future studies might be helpful.

### 4.3. Some functional considerations

Juxtaposed to the outer retina, the choroid has many complex roles in retinal and ocular physiology, (Liang et al., 2004; Lutty and McLeod, 2018; Nickla and Wallman, 2010) at least in part mediated by its dense vasculature and its rich sensory/autonomic innervation and intrinsic neurons. Besides the long-appreciated function of choroidal blood flow in retinal health, other presumed roles of the choroid include regulation of refractive development and eye growth, thermoregulation, and mediation of light-induced nociception. The roles of OPN4 in such ocular functions are under active investigation, and the complexity of the cellular localizations of OPN4 here support the notion that OPN4 has multiple functions within the choroid.

Post-natal ocular growth and refraction are now thought to be regulated by visual image quality through a pathway from retina to choroid to sclera. This pathway presumably involves signaling molecules transported to or across the choroid (Liu et al., 2021; Zhang and Wildsoet, 2015). In addition, the choroid undergoes circadian oscillations in its thickness that may also modulate scleral and ocular growth (Nickla, 2013). Numerous tissues, many not even exposed to light normally, express opsins that are affected by light (Moraes et al., 2021). Given the distribution of OPN4 found here, the effects of altered visual input on OPN4 expression in the chick choroid (Stone et al., 2020) and the refractive effects of reduced retinal OPN4 activity in mice (Chakraborty et al., 2022), it would appear imperative that the nature of the role of choroidal OPN4 be sought in refractive development.

It has long been postulated that the high rate of choroidal blood flow could act as a heat source or sink that protects the retina from extreme changes in temperature (Parver, 1991; Parver et al., 1980, 1982). Based on work in skin (Moraes et al., 2017, 2021), OPN4 seemingly acts as a thermo-detector that links heat to expression levels of the clock genes *Bmal1* and *Per1*. Whether OPN4 acts as a thermo-detector in choroid requires direct study, while the structures provided here, by localization and type, could contribute (Schrodl et al., 2003).

As another example pertinent to the neural localizations here, OPN4 has been identified in trigeminal neurons and nerve fibers projecting to the eye. (Delwig et al., 2018; Matynia et al., 2016). These reports differ on whether these OPN4 containing neurons sense light. Even so, the co-localization here of OPN4 with the sensory neuropeptide substance P supports the notion that OPN4 in the choroid has some neural function meriting further investigation.

In summary, the choroid of both human and chicken contains nerve fibers and cells that are immunoreactive for OPN4. Some of the cells are morphologically similar to intrinsic choroidal neurons, and in human choroid, some are melanocytes. We propose that these OPN4-containing structures mediate developmental, light-, thermally-driven, and/or nociceptive phenomena in the eye, hypotheses that suggest directions for investigating novel functions of the choroid.

## Figures and Tables

**Table 1:** primary antibodies used in this study.

antigen	abbreviation	host	type	dilution	supplier (Cat #)
Melanopsin	OPN4	rabbit	polyclonal; immunogen affinity purified	1:200	Novus Biologicals, Abington, UK (NB100-74460)
Vasoactive intestinal peptide	VIP	guinea-pig	Polyclonal; whole antiserum	1:1000	Progen, Heidelberg, Germany (16071)
Macrosialin/CD68	CD68	mouse	monoclonal	1:100	Dako, Glostrup, Denmark (M087601-2)
Substance P	SP	guinea-pig	polyclonal; whole antiserum	1:1000	Novus Biologicals, Abington, UK (NB300-187)
α-smooth muscle actin	ASMA	mouse	monoclonal	1:5000	Sigma-Aldrich, Vienna, Austria (A2547)

## Data Availability

Data will be made available on request.
